# Randomised controlled field study to evaluate the efficacy and clinical safety of a single 8 mg/kg injectable dose of marbofloxacin compared with one or two doses of 7.5 mg/kg injectable enrofloxacin for the treatment of *Actinobacillus pleuropneumoniae* infections in growing-fattening pigs in Europe

**DOI:** 10.1186/s40813-017-0057-2

**Published:** 2017-05-10

**Authors:** Erik Grandemange, Pierre-Alexandre Perrin, Dejean Cvejic, Miriam Haas, Tim Rowan, Klaus Hellmann

**Affiliations:** 1 Vetoquinol SA, Research and Development Centre, B.P. 189, 70204 Lure Cedex, France; 2 Klifovet AG, Geyerspergerstr 27, D-80689 Munich, Germany; 3 Rowdix Ltd, Folly Hall, Cawton, York, YO62 4LW UK

**Keywords:** Marbofloxacin, Enrofloxacin, Respiratory disease, Efficacy, *Actinobacillus pleuropneumoniae*, Minimum inhibitory concentration

## Abstract

**Background:**

Acute outbreaks of *Actinobacillus pleuropneumoniae* (APP) require rapid, effective, parenteral antimicrobial treatment. The efficacy and safety of a single, short-acting, high dose of marbofloxacin (Forcyl® swine 160 mg/mL) compared with 1 or 2 doses of 7.5 mg/kg enrofloxacin in APP outbreaks in European farms was studied.

**Methods:**

A controlled, randomised block, blinded, multicentre, field study was conducted on four farms with acute respiratory disease associated with APP. Animals with clinical signs of respiratory disease were allocated similarly to intramuscular treatments of either a single dose 8 mg/kg marbofloxacin on day 0 or, 7.5 mg/kg enrofloxacin (Baytril 1nject®) on day 0 and again on day 2, if clinical signs had not improved.

**Results:**

The results were similar for intention to treat (242 pigs) and per protocol populations (239 pigs). On day 0, all pigs had pyrexia (means, 40.6 °C), moderate to severe clinical signs (depression, cough, dyspnoea). Following treatment, animals improved rapidly and on day 7, clinical signs were absent or mild in all pigs and mean temperatures for each treatment were <39.5 °C (*P* > 0.05). The primary efficacy criterion, animals cured, for marbofloxacin and enrofloxacin was 81.8 and 81.4% on day 7, and 84.2 and 82.2% on day 21, respectively. Results for cure, respiratory disease removals and mortalities, and relapses were compared using confidence intervals and confirmed that marbofloxacin was non-inferior to enrofloxacin (*P* > 0.05). There were no significant treatment differences in live weight gains, adverse events and injection site reactions (<2.5% animals) (*P* > 0.05). Significantly more animals developed concurrent disorders in the enrofloxacin (7.5%) than marbofloxacin (0.0%) group (*P* < 0.01). On day 0, the MIC_90_ values of APP for marbofloxacin and enrofloxacin were 0.06 μg/mL for APP, less than the clinical breakpoints.

**Conclusions:**

Marbofloxacin (single dose of 8 mg/kg) and enrofloxacin (1 or 2 doses of 7.5 mg/kg) were clinically safe and effective in the treatment of clinical respiratory disease associated predominantly with APP in four European commercial, fattening pig herds.

## Background

Respiratory infections are a common cause of morbidity and mortality in growing-fattening pigs [1, 2]. Even on well-managed pig farms with effective prevention systems, pigs may become infected with bacterial pathogens requiring antimicrobial treatment. Common porcine, bacterial respiratory pathogens include *Actinobacillus pleuropneumoniae* (APP), *Pasteurella multocida* (PM), *Haemophilus parasuis, Mycoplasma hyopneumoniae* and *Bordetella bronchiseptica* [2, 3]. APP is found worldwide and is an important pathogen of pigs. Clinical signs are most common in pigs less than 6 months of age and include acute, life-threatening, pleuropneumonia with coughing, fever and lethargy. At post mortem lesions there may be necrotic and haemorrhagic lung lesions with lung tissue sequestration and fibrotic pleuritis and pericarditis. Infected but clinically healthy animals carrying the pathogen are an important source of the infection [1–3]. Reduced production efficiency including decreased daily weight gain, reduced feed conversion, carcass trimming and condemnation, and costs and time for intensive animal treatment [1, 2] may be associated with APP infections.

The treatment of acute APP infections is essential to ensure animal welfare and will contribute to minimising adverse economic effects. Selection of an antimicrobial treatment by a field veterinarian, animal owner or farm staff requires consideration of efficacy, dosage regimen and formulation based on the product label including precautions to minimise the potential for resistance development [2, 3]. Minimising use of antimicrobials is widely recognised as important to restricting the development of antimicrobial resistance and this favours the treatment of individual, clinically affected animals rather than administration through feed or water involving treatment of an entire group of pigs containing the clinically affected animals. Furthermore, for acutely diseased pigs that are unlikely to eat and perhaps drink in severe cases it is essential to administer antimicrobials to individual animals, for example by parenteral injection. An individual animal treatment which only requires a single administration is desirable to minimise animal handling and to facilitate compliance with the recommended dosing regimen by farm staff. However, many antimicrobial treatments require at least daily administration for three or more days [2, 4, 5].

The fluoroquinolone antimicrobials are unusual in that their safety and concentration-dependent mode of action facilitates the use of a single, short-acting, high dose to provide both good efficacy and to minimise resistance development in target pathogens such as APP [6, 7]. Analysis based on antimicrobial pharmacokinetics (PK) in growing-fattening pigs and on pharmacodynamics (PD), using both minimum inhibitory concentrations (MIC) and mutation prevention concentrations (MPC) of representative field isolates of APP, PM and *H. parasuis* has provided a strong rationale for field use of a single, IM dose of 8 mg/kg marbofloxacin in the treatment of acute actinobacillosis and which is also unlikely to favour resistance development in the target pathogens [8–12]. However, fluoroquinolones are classified as critically important antimicrobials for human health and in order to minimise resistance development their veterinary use should be reserved for the treatment of clinical conditions which have responded poorly, or are expected to respond poorly to other classes of antimicrobials. Wherever possible, use of a fluoroquinolone product should only occur when based on relevant epidemiological data and susceptibility testing (preferably specific to the affected herd), and after consideration of alternatives, local antimicrobial usage policies and the absence of an alternative and effective non-critically important antimicrobial [13–15]. Improvements to husbandry practices and vaccination should also be considered to reduce the risk of future disease outbreaks [14].

The use of studies with negative (untreated) controls to evaluate efficacy of antimicrobials is limited for ethical and animal welfare reasons. A well-controlled, in vivo study based on an aerosol challenge infection of APP in pigs showed that a single IM dose of 8 mg/kg marbofloaxcin was at least as effective as a positive control of conventional daily dosing for three days with enrofloxacin (2.5 mg/kg/ day; total dosage 7.5 mg/kg/animal) and superior to a negative, saline control treatment (clinical cures 6 days after infection were 91, 83 and 9%, respectively) [16]. The study reported here was to determine whether or not similar efficacy of a single 8 mg/kg dose of marbofloxacin would be obtained under field conditions of natural outbreaks of APP clinical disease in pigs. Some preliminary results of the study were presented previously [17].

## Methods

The study treatments are shown in Table 1. The distribution of animals to study sites (farms) and treatment groups, and clinical scores and rectal temperatures before treatment on day 0 (per protocol population) are shown in Table 2. The distribution by farm of bacterial pathogens isolated before treatment on day 0 from lower respiratory tract lesions and bronchoalveolar lavage samples, and ranges of minimum inhibitory concentrations (MIC) of marbofloxacin and enrofloxacin are shown in Table 3.

**Table 1:** Study treatment groups (dosages and duration of treatments are as recommended in product data sheets at the time of the study)

Treatment group (Active ingredient)	Route of administration	Dosage mg/kg	Duration of treatment	Day(s) of observation	Number of animals^a^
Marbofloxacin	IM	8	Once on day 0 only	0, 1, 2, 3, 7, 21	122
Enrofloxacin	IM	7.5	Once on day 0 & on day 2 if clinical signs had not improved	0, 1, 2, 3, 7, 21	120

^a^Intention to treat populations

**Table 2:** Distribution of animals by farm and treatment group, and homogeneity of clinical scores and rectal temperatures before treatment on day 0 (per protocol population)

		Marbofloxacin (T1)				Enrofloxacin (T2)				T1 vs. T2 *P* value^a^
	Farm site	Total no. animals	Mild	Moderate	Severe	Total no. animals	Mild	Moderate	Severe	
Respiratory scores % [n]^b^	GA	36	0.0 [0]	97.2 [35]	2.8 [1]	35	0.0 [0]	100 [35]	0.0 [0]	0.324
	GB	35	0.0 [0]	82.9 [29]	17.1 [6]	35	68.6 [24]	31.4 [11]	0.0 [0]	0.166
	HA	24	0.0 [0]	100 [24]	0.0 [0]	24	0.0 [0]	100 [24]	0.0 [0]	nc
	HB	26	0.0 [0]	100 [26]	0.0 [0]	24	0.0 [0]	95.8 [23]	4.2 [1]	0.298
	Combined	121	0.0 [0]	94.2 [114]	5.8 [7]	118	0.0 [0]	89.8 [106]	10.2 [12]	0.193
Depression scores % [n]^b^	GA	36	97.2 [35]	2.8 [1]	0.0 [0]	35	97.1 [34]	2.9 [1]	0.0 [0]	0.984
	GB	35	85.7 [30]	11.4 [4]	2.9 [1]	35	85.7 [30]	14.3 [5]	0.0 [0]	0.961
	HA	24	70.8 [17]	29.2 [7]	0.0 [0]	24	83.3 [20]	16.7 [4]	0.0 [0]	0.308
	HB	26	100 [0]	0.0 [0]	0.0 [0]	24	95.8 [23]	4.2 [1]	0.0 [0]	0.298
	Combined	121	89.3 [108]	9.9 [12]	0.8 [1]	118	90.7 [107]	9.3 [11]	0.0 [0]	0.647
Rectal temperatures °C	GA	36		40.4 (0.047)		35		40.4 (0.049)		0.702
	GB	35		40.6 (0.068)		35		40.6 (0.069)		0.556
	HA	24		40.6 (0.114)		24		40.4 (0.060)		0.737
	HB	26		40.7 (0.077)		24		40.9 (0.091)		0.240
	Combined	121		40.6 (0.037)		118		40.6 (0.034)		0.603

Farm site: Country identification *G* Germany, *H* Hungary. A and B indicate different farms within country

Numbers of animals and percentages refer to the per protocol populations. Animals euthanised prior to study for diagnostic purposes are excluded from these populations

^a^For respiration and depression scores, *p* values are from Mantel-Haenszel mean score for each site separately and on Cochran-Mantel-Haenszel mean score statistic for combined

^b^Refer to Clinical examinations for details of scoring. [n] number of animals. nc Not calculated

Rectal temperatures shown as mean (standard error). For rectal temperatures, *p* values are based on Wilcoxon rank sum statistic for each site separately and on the extended Mantel-Haenszel statistic for combined

**Table 3:** Distribution by farm of bacterial pathogens isolated before treatment on day 0 from lower respiratory tract lesions and bronchoalveolar lavage samples, and ranges of minimum inhibitory concentrations (MIC) of marbofloxacin and enrofloxacin

		MIC range (μg/mL)		
	Farm site	Marbofloxacin	Enrofloxacin	*n*
*A. pleuropneumoniae*	GA	0.03–0.06	0.06–0.12	9
	GB	0.03–0.25	0.03–0.25	9
	HA	0.06–0.12	0.06–0.12	18
	HB	0.03–0.06	0.03–0.06	25
*H. somnus*	GA	–	–	0
	GB	0.015	0.008	1
	HA	–	–	0
	HB	0.03	0.03	1
*P. multocida*	GA	–	–	0
	GB	0.015	0.008	1
	HA	0.015–0.03	0.008	7
	HB	–	–	0
*B. bronchiseptica*	GA	0.5	0.5	4
	GB	0.25–0.5	0.25–0.5	4
	HA	–	–	0
	HB	0.25	0.5	1

Farm site: Country identification *G* Germany, *H* Hungary. A and B indicate different farms within country

MIC concentrations ranges (μg/mL) of the antimicrobial for which all of the isolates were inhibited

*n* total number of isolates from lung/lower respiratory tract and bronchoalveolar lavage, combined, for which MIC was determined

### Herd description, study animals, management

Pig fattening units on each of four farms (two in each of Germany (G) and Hungary (H)) were selected for the study based on history of swine respiratory disease (SRD) associated with APP confirmed by necropsy and bacteriology in the previous three months. Three of the farms (HA, HB, GA) included breeding and fattening pig units, and one farm (GB) was of fattening pigs only. Within each farm, pigs were obtained from a single source. Pig population sizes for each farm were 1600, 6500, 9500 and 13,000 for farms GB, GA, HB, and HA, respectively. Study animals were housed on fully (HB, GA, GB) or partially (HA) slatted floors with negative pressure ventilation and, in three farms (HA, HB GA), temperature controlled environments. Commercial feed was given either *ad libitum* (HA, HB, GB) or restricted (GA) as meal (GA, GB) or pellets (HA, HB)), and water by nipple drinkers. Breeds were Hungarian Large White x Hungarian Landrace x Duroc (HA), European hybrid (HB), BHZP Viktoria and BHZP Naima (GA) and Topigs (GB). Routine vaccinations were used on each farm, including for prevention of the following infections: PCV2 and *Mycoplasma hyopneumoniae* (HA, HB, GB) and PRRSV (HA, GA, GB). A total of 242 fattening, male (mostly castrates) and female pigs, weighing 25 to 94 kg live weight, and between 15 and 20 weeks of age were enrolled in the study.

Administration of vaccines was completed at least 3 weeks before first treatment (day 0); administration of anti-inflammatory and other antimicrobial products was not permitted in the study and for 7 days (or 21 days for long-acting products) prior to day 0.

### Study design

The objectives of the study were to determine the clinical efficacy and clinical safety under field conditions of IM administration of a single dose of 8 mg/kg marbofloxacin (160 mg/mL, Forcyl® Swine, Vetoquinol SA, France) compared with one or two IM administrations of 7.5 mg/kg enrofloxacin (100 mg/mL, Baytril 1nject®, Bayer Vital GmbH, Germany) against naturally-occurring respiratory disease associated principally with APP in pigs on commercial farms in Europe. The control product, enrofloxacin was chosen as a reference or control product as it, like marbofloxacin is a second generation fluoroquinolone and has similar pharmacokinetic properties, is indicated for the treatment of APP in pigs and may be used for single dose therapy. It should be noted that the dosage regimen for enrofloxacin in the treatment of SRD in the study was according to the previous summary of product characteristics, and that this has since changed to a single dose of 7.5 mg/kg only and the current summary of product characteristics does not refer to administration of a second dose [13]. The study design was a positive controlled, randomised and blocked, blinded, multicentre, clinical field study with natural infection. The experimental and treatment unit was the individual animal. There were four farms in the study and the study was designed and powered to compare the treatments using the combined data from the four farms (Table 2). The distribution and sensitivity range of the isolates from the lower respiratory tract were also similar between farms on day 0 (Table 3). The study was not designed, and did not have power to make conclusions based on any apparent differences in results between farms and such difference may be confounded with other, uncontrolled variables (e.g. housing, diet, management and genotype).

Study day 0 was defined individually for each animal as the day it first met the clinical criteria for treatment and was first administered either the investigational veterinary product, marbofloxacin, or the positive control product, enrofloxacin. The primary efficacy criterion was animals cured clinically on day 7 and the power of the study was based on this criterion. A minimum of 112 clinically affected, per protocol animals were required for each treatment group to provide statistical power of 80% and a non-inferiority margin of 0.15 (15 percentage points) based on known efficacy of approximately 80% for the control product, an anticipated placebo or self-cure response of 46% [18] and a 5% significance level. Animals were enrolled according to pre-defined inclusion and exclusion criteria and were allocated equally to each treatment group on day 0. On day 0, animals were injected IM with the either marbofloxacin or enrofloxacin on either the right hand side (farms HA and HB) or the left hand side (farms GA and GB) of the neck. For the enrofloxacin treatment group only, a second administration approximately 48 h later was permitted if clinical signs had not improved, as assessed by the blinded examining veterinarian using the same pre-defined criteria as applied on day 0.

The study was conducted according to good clinical practice [19] by four, separate investigators with independent monitoring by a contract research organisation (Klifovet AG, Germany). To facilitate quality and consistency across the study sites or farms, all staff received specific training for the study including, as appropriate necropsy examinations, sample collection and clinical examination scoring. The conduct of the study was monitored at all stages by an appointed individual that was independent of the investigators and veterinarians, and responsible for overseeing the clinical study and ensuring that it was conducted, recorded, and reported in accordance with the study protocol, standard operating procedures, Good Clinical Practice and the applicable regulatory requirements. This included on-site inspections during necropsies, product administration and clinical examinations. There were different investigators, examining veterinarians and dispensers for each farm. The investigators and examining veterinarians could be the same individual and were specialised veterinary pig practitioners, experienced in both clinical and post mortem examinations. The treatments were administered by the dispensers (the individual farm veterinarians) following a pre-established randomisation plan. The study was blinded by using different personnel: All personnel making clinical, post mortem and laboratory examinations, and decisions to treat (including any decision to administer a second treatment of enrofloxacin) or to withdraw animals from the study were blinded (and remained blinded until the end of the study) to the allocation of animals to treatment; different, unblinded personnel were used for administration of treatments. A single, centralised laboratory was used for all microbiological isolation and PCR analyses (University of Veterinary Medicine, Hannover, Germany) and another laboratory (Vetoquinol SA, Lure , France) was used for all determinations of MIC. All laboratory staff were blinded. The in vivo phase of the study was conducted between November 2013 and April 2014. Statistical analyses, accountability of marbofloxacin and enrofloxacin use, and quality assurance procedures were conducted by independent staff.

### Variables for efficacy and safety assessments

#### Clinical examinations and live weights

Animals were examined by the blinded, examining veterinarians on days 0, 1, 2, 3, 7 and 21. Rectal temperatures and clinical assessments (scores) of respiratory signs, depression and injection site reactions were recorded. For an animal to be enrolled on day 0, it was required to have a temperature of ≥40.2 °C, and a minimum of moderate respiratory signs and moderate depression as determined by the blinded examining veterinarian. Respiratory signs were assessed as normal, mild (mild dyspnoea and/or cough, little or no nasal discharge), moderate (moderate dyspnoea with multiple episodes of coughing within a few minutes and nasal discharge) or severe (coughing, nasal discharge, and gasping or open mouthed breathing and/or cyanosis). Signs of depression was assessed as absent, mild (slight depression, active but not fully alert, reduced appetite), moderate (obvious depression, responded only after stimulation, head down, anorexia) or severe (not eating, no response to stimulation, unable to stand, and moribund). Animals showing severe respiratory signs or severe depression were considered for immediate euthanasia. Normal rectal temperature was defined as ≤40 °C based on preliminary clinical examinations and rectal temperature recordings of pigs at each farm prior to the APP outbreaks. Injection site reactions were recorded as reddening, swelling, induration and pain on palpation.

The primary and secondary efficacy criteria were animals cured on day 7 and day 21, respectively. Cure was defined as normal rectal temperature (≤40 °C) and absence of clinical signs of depression and absence of respiratory signs. Any animals removed from the study was assigned a reason for removal and if appropriate necropsied. Animals removed because of SRD before day 7 were counted as not cured. Animals removed from the study were either given alternative treatment or euthanised for animal welfare reasons.

Live weights of animals were recorded on days 0 and 21.

#### Post mortem and laboratory examinations

The aetiology of each SRD outbreak was established by necropsy of pigs within 1 h of euthanasia (or natural mortality) of up to 10 pigs per farm in the 24 h before first treatment administration. These pigs were not part of the intention to treat population; all other pigs on each farm that met the inclusion criteria on a single day (i.e. day 0 which was a calendar date specific to each farm) were included in this population. Each pig selected for necropsy had clinical signs of SRD. The numbers of pigs selected for necropsy (and the numbers of live animals enrolled in the study) for farms GA, GB, HA and HB were 4 (and 71), 10 (and 70), 6 (and 50), and 10 (and 51), respectively. To assist the rapid and consistent collection of samples for bacteriology, necropsies were performed to a standard procedure on each farm in a designated area. In addition, necropsies were made from days 1 to 21 on five animals either removed from the study and euthanised on welfare grounds or after natural mortality. Broncho-alveolar lavage samples (BAL) [20] for bacteriology were collected from another 36 pigs (maximum of 10 animals / farm) before treatment on day −1 or 0 and again from the same animals on day 7. Nasal swabs (single nostril, approximately 10 cm deep) were collected on days 0 and 7 from all live animals that were not sampled by BAL.

A total of 369 bacterial isolates (APP, *B. bronchiseptica, H. parasuis* and PM), were obtained either from lungs at necropsy or, from live animals by BAL or nasal swabbing and were identified by culture and PCR [21–27]. The isolates were used for MICs determinations by standard, broth microdilution, procedures of Vetoquinol laboratories (with adherence to the Clinical and Laboratory Standards Institute (CLSI) guidelines [28–30]) except for *H. parasuis* where veterinary fastidious medium was used as described for APP in the CLSI standard VET01-A4 [28]. The MIC determinations included standard reference (quality control) strains of *Staphylococcus aureus* ATCC 29213 and *Escherichia coli* ATCC 25922, and sterility and growth controls on each plate. The MICs of the quality control strains were within the CLSI quality control ranges. Where 10 or more isolates of a bacterial species were available, the MIC_50_ and MIC_90_ values (concentration of antimicrobial required to inhibit 50 and 90%, respectively, of a population of isolates of the same species) were calculated and then rounded up to the next value in standard antimicrobial sensitivity test dilution series [31].

On farms HA and GA only, blood samples for serology were collected from 10 pigs selected randomly from within the per protocol populations on day 0 and these pigs were resampled on day 21. These samples were not an integral part of the study and were collected to assist in the diagnosis of possible intercurrent disease if it should have arisen during the study. Samples were analysed for seroconversion to PRRS virus (ELISA; PRRS X3 Ab test, IDEXX), PCV-2 virus (Capture-ELISA; INgezim Circovirus IgM/IgG, INGENASA) and Influenza A virus (H1N1, H1N2, H3N2, H1N1 haemagglutination test, IDT Biologika).

### Statistical analyses

The intention to treat population of animals was a total of 242 (Table 1) and of these, three animals were excluded from the per protocol population because of concomitant disorders not related to SRD which did not allow the animals to continue in the study as other treatments were required that were not compliant with the protocol. The per protocol population was a total of 239 animals (Tables 2 and 4). Safety evaluations used all of the 242 animals that were enrolled in the study on day 0 (intention to treat population) and the per protocol population of 239 animals was used for efficacy evaluations. Homogeneity criteria of per protocol populations on day 0 for age, sex, weight, respiratory and depression scores, and rectal temperature were confirmed to be similar between treatments (*P* > 0.05). Animals in each treatment group were mixed together within each pen on each farm and the individual animal was the experimental unit. Comparisons of animals cured clinically on days 7 and 21, were made by non-inferiority analyses for marbofloxacin compared with enrofloxacin and the null hypothesis was rejected if the lower bound of the 95% confidence interval was greater than 0.15 (corresponding to a 15% difference in percentages of animals cured; Fisher’s exact test, two-tailed). Animals removed because of SRD before day 7 were counted as not cured. Non-inferiority analyses were also used for comparisons between treatments of animals that were (i) mortalities or removed for SRD, and (ii) relapsed with SRD from days 8 to 21 inclusive. Cochran-Mantel-Haenszel chi-squared statistics (adjusted where appropriate for farm) and confidence intervals (CI) were used to confirm similarity of treatment groups before treatment administration on day 0, and following treatment to compare differences in respiratory and depression scores between treatment groups. Reductions in rectal temperatures from day 0 to day 7 were compared by repeated measures of analysis of variance and 95% confidence limits for least squared means. Live weights, adjusted for day 0 values, were compared by analysis of variance. Percentages of animals in each treatment group with concurrent disorders, injection site reactions, adverse events (any observation in animals that was unfavourable and unintended and occurred after the use of an investigational veterinary or control product, whether or not considered to be product related) and suspected adverse drug reactions were compared using Fisher’s exact test. Statistical significance of difference was obtained when *P* < 0.05 (two-tailed). Data was validated by the double data entry method. All statistical calculations were performed using SAS® 9.3 software programmes (SAS Institute Inc, Cary, North Carolina, USA).

**Table 4:** Clinical efficacy and safety results for marbofloxacin and enrofloxacin in the treatment of APP associated respiratory disease in fattening pigs

		Marbofloxacin				Enrofloxacin				CI of treatment difference
		Absent	Mild	Moderate	Severe	Absent	Mild	Moderate	Severe	
Respiratory scores % [n]^a^	Day 0	0.0 [0]	0.0 [0]	94.2 [114]	5.8 [7]	0.0 [0]	0.0 [0]	89.8 [106]	10.2 [12]	
	Day 2	43.0 [52]	53.7 [65]	2.5 [3]	0.8 [1]	46.2 [54]	49.6 [58]	4.3 [5]	0.0 [0]	
	Day 3	68.6 [83]	28.1 [34]	3.3 [4]	0 [0]	65.8 [77]	32.5 [38]	1.7 [2]	0 [0]	
	Day 7	94.2 [113]	5.8 [7]	0.0 [0]	0.0 [0]	93.2 [109]	6.8 [8]	0.0 [0]	0.0 [0]	
	Day 21	94.7 [108]	3.5 [4]	1.8 [2]	0.0 [0]	95.7 [110]	4.3 [5]	0.0 [0]	0.0 [0]	
Depression scores % [n]^a^	Day 0	0.0 [0]	89.3 [108]	9.9 [12]	0.8 [1]	0.0 [0]	90.7 [107]	9.3 [11]	0.0 [0]	
	Day 2	59.5 [72]	38.0 [46]	2.5 [3]	0.0 [0]	59.8 [70]	40.2 [47]	0.0 [0]	0.0 [0]	
	Day 3	80.2 [97]	18.2 [22]	1.7 [2]	0 [0]	80.3 [94]	19.7 [23]	0 [0]	0 [0]	
	Day 7	90.8 [109]	9.2 [11]	0.0 [0]	0.0 [0]	92.3 [108]	7.7 [9]	0.0 [0]	0.0 [0]	
	Day 21	97.4 [111]	2.6 [3]	0.0 [0]	0.0 [0]	97.4 [112]	2.6 [3]	0.0 [0]	0.0 [0]	
Rectal temperatures °C	Day 0	40.6 (0.41) [121]				40.6 (0.40) [118]				
	Day 2	39.6 (0.42) [121]				39.7 (0.36) [117]				−0.05; 0.15
	Day 3	39.6 (0.49) [121]				39.6 (0.56) [117]				−0.016; 0.11
	Day 7	39.4 (0.52) [120]				39.4 (0.51) [117]				−0.12; 0.14
	Day 21	39.4 (0.43) [114]				39.5 (0.42) [115]				0.01; 0.23
Retreatment % animals^b^	Day 2	n/a				17.8 [21]				
Clinical cure % animals^b^	Day 7	81.8 [99]				81.4 [96]				−9.37; 10.29
	Day 21	84.2 [101]				82.2 [97]				−7.54; 11.47
SRD removals & mortalities %^b^	Days 0–21	5.0 [6]				2.5 [3]				−7.22; 2.38
SRD relapses % animals^b^	Days 8–21	0.0 [0]				1.0 [1]				−0.99; 3.07
Adverse events % animals (CI)^c^		4.1 [5]				6.7 [8]				−3.1; 8.3
Injection site reactions % animals^c^		2.4 [3]				1.6 [2]				
Concurrent disorders % animals^c^		0.0^d^ [0]				7.5^e^ [9]				
Live weight gain kg	Days 0–21	19.81 (5.06) [114]				20.05 (7.03) [115]				−0.026; 0.109
No. animals: Intention to treat^c^	Day 0	122				120				
No. animals: Per protocol^b^	Day 7	121				118				

Results are for the overall study which included 4 farms and are shown as mean (standard deviation) unless indicated otherwise; *CI* confidence interval

^a^Refer to Clinical examinations for detailed description of individual scores. [n] number of animals

^b^Indicated efficacy data percentages are based on per protocol populations. Note that percentages for respiratory and depression scores, and rectal temperatures are based on numbers of animals in a treatment group on a given day, [n]

^c^Indicated safety data percentages are based on intention to treat populations. There were no suspected adverse drug reactions

^d,e^values in a row are significantly different. *n/a* Not applicable

Animals euthanised prior to study for diagnostic purposes are excluded from each of the populations

### Ethical and animal welfare approvals

The study was a clinical field trial conducted using naturally occurring cases of SRD, treated with approved veterinary prescription products and was conducted under detailed and direct veterinary supervision. The study was reviewed and approved by an ethical and animal welfare committee (Klifovet reference number: 01449-009-1). The owners of the farms in the study gave their written informed consent. The study was conducted according to European regulatory requirements and in compliance with German and Hungarian drug and animal welfare legislation, and with European Medicines Agency guidelines for demonstration of efficacy of veterinary medicinal products containing antimicrobial substances and for statistical principles in veterinary clinical trials [32, 33]. Animals were excluded or removed from the field trial if the severity of the clinical signs indicated, in the view of the attending veterinarians, that euthanasia (or withdrawal from the study) was most appropriate. Animals intended for treatment and removed from the study are reported in the results.

## Results

The clinical results are shown in Table 4. The study comprised four outbreaks of APP, one from each of the four farms, and each outbreak of APP was the first that occurred on each farm beginning from November 2013. For the marbofloxacin and enrofloxacin treatments, there were 122 and 120 pigs in the intention to treat populations, and 121 and 118 pigs in the per protocol populations, respectively. Three animals were removed from the study prior to treatment administration on day 0 for reasons not related to SRD (one pig allocated to each of the treatment groups died during blood sampling and one pig allocated to the enrofloxacin group was euthanised for welfare reasons and at necropsy had extensive pulmonary haemorrhages). Of 36 pigs (up to 10 pigs per farm) examined by necropsy prior to treatment administration, 30 had both gross lesions of pleuropneumonia and moderate to heavy growth of APP on culture (pure cultures on NAD supplemented blood agar, confirmed by PCR; serotypes 2 (farms HB, GA, GB) and 7 (HA)). Six of the 36 animals did not have typical lesions and samples were not collected for bacteriology from these animals.

The outbreaks of APP were acute and 100% of enrolled pigs on day 0 showed moderate to severe respiratory clinical signs and, moderate signs of depression in each of the treatment groups (*P* > 0.05; one animal on day 0 was unintentionally included with severe depression). On day 0, all enrolled animals were pyrexic with mean rectal temperatures of 40.6 °C in both treatment groups (*P* > 0.05). Following antimicrobial treatment on day 0, there were rapid clinical responses in both treatment groups (*P* > 0.05) and by day 2 there were marked reductions in the prevalence and severity of pigs with moderate or severe respiratory signs and/or depression, and mean rectal temperatures were reduced to 39.6 and 39.7 °C for the marbofloxain and enrofloxacin groups, respectively (*P* > 0.05). However, on day 2, 21 animals (17.8%) in the enrofloxacin group distributed across three of the farms (HA, HB and GA) were assessed by the blinded examining veterinarians to still have clinical signs that had not improved (i.e. signs were the same or of greater severity than on day 0) and therefore, following the recommendations of the previous Summary of Product Characteristics, a second administration of enrofloxacin was given to these animals. (Note that the dosage regimen for enrofloxacin in the treatment of SRD is now for a single dose of 7.5 mg/kg only) [13]. In the marbofloxacin group on day 2, one animal (0.8%) showed severe respiratory signs but it did not have signs of depression and was allowed to continue in the study (without additional treatment) by the blinded examining veterinarian. Overall in each of the treatment groups, animals continued to improve compared with day 0 and by one week after treatment, the percentages of animals with either no respiratory signs and/or no depression were >90% and mean rectal temperatures were normal (*P* > 0.05).

Efficacy as indicated by clinical cure on day 7 was apparent in 99 (81.8%) and 96 (81.4%) of animals in the per protocol marbofloxacin and enrofloxacin groups, respectively. Results for both treatment groups were consistent and similar between the per protocol and intention to treat analyses. The difference in percentages of animals cured on day 7 for marbofloxacin – enrofloxacin were +0.4% for the per protocol population and +1.8% for the intention to treat population. Non-inferiority of marbofloxacin compared with enrofloxacin was shown, and the mean cure rates on day 7 were similar (*P* > 0.05). Inferiority, the null hypothesis, would have been rejected if the lower bound of the 95% confidence interval of the difference in the percentage of animals cured on day 7 was greater than −15%. At the end of the study, day 21, the clinical cure for marbofloxacin was 84.2% and non-inferior to that for enrofloxacin, 82.2% (*P* > 0.05). From day 0 to day 7, and from day 8 to day 21 the SRD removals and mortalities, and relapses of animals in each treatment were similar and non-inferior for marbofloxacin compared with enrofloxacin (*P* > 0.05).

Serological assays on the blood samples collected on days 0 and 21 from samples of pigs in the per protocol populations on farms GA and HA found no evidence of seroconversion to PRRSV, PCV2 or swine influenza viruses. This suggests that there were no clinical disease outbreaks of these viral infections in the three-week study period on farms GA and HA.

Adverse events in the present study occurred similarly in the marbofloxacin and enrofloxacin groups (4.1 and 6.7%, respectively; *P* > 0.05) and were considered by the each of the investigators as unlikely to have been related to the antimicrobial treatments (i.e. there were no suspected adverse drug reactions attributed to either marbofloxacin or enrofloxacin). Adverse events included inflammation of the pinna associated with ear tagging, death associated with blood sampling, diarrhoea and lameness. Injection site reactions of limited swelling and, or pain of short duration (<3 days) occurred in 2.46 and 1.67% of the marbofloxacin and enrofloxacin groups, respectively (*P* > 0.05). Concurrent disorders (lameness, diarrhoea, swollen leg, abscess, hernia, tail bite and haematoma) beginning after enrolment on day 0 were observed in 9 or 7.5% of animals in the enrofloxacin group (3, 1, 5 and 0 animals for farms GA, GB, HA and HB, respectively) significantly more than 0% in the marbofloxacin group (*P* < 0.01). In the marbofloxacin and enrofloxacin groups, the mean live weights on day 0, 56.3 and 55.5 kg, respectively, and the mean live weight gains from day 0 to day 21, 19.81 and 20.05 kg, respectively, were similar (*P* > 0.05).

The MIC and susceptibility results for isolates obtained from the four farms in the study are shown in Tables 3 and 5. Determination of MICs for marbofloxacin and enrofloxacin were successfully made on a total of 361 and 352 isolates, respectively. The majority of the isolates were from day 0 (pre-treatment) and day 7, and comprised 66 APP, 50 PM, 20 *H. parasuis* and 230 *B. bronchiseptica* isolates. Sixty-four APP isolates, eight PM*,* two *H. parasuis* and 22 *B. bronchiseptica* isolates were from lung and BAL samples; the remaining isolates were from nasal swabs. Sixty-one isolates of APP were obtained on day 0, and, following treatment, no APP were isolated on day 7 by BAL; five APP isolates were obtained from lungs at necropsy of animals removed from the study on days 1 and 15. The MIC_90_ values for the 66 APP isolates were 0.06 μg/mL for marbofloxacin and enrofloxacin. Clinical susceptibility breakpoints for APP and PM have been published by CLSI for enrofloxacin [29] and by CASFM (Comité de l’antibiogramme de la société Française de Microbiologie) for marbofloxacin [30]. All of the APP isolates were susceptible to both marbofloxacin and enrofloxacin (clinical breakpoint ≤0.25 μg/mL; resistance ≥1 μg/mL for enrofloxacin and 1 and 2 μg/mL respectively for marbofloxacin). Thirty-two isolates of PM were obtained on day 0 and the MIC_90_ values were 0.03 and 0.015 μg/mL for marbofloxacin and enrofloxacin, respectively. As for APP, all of the PM isolates were also susceptible to both antimicrobials (clinical breakpoint ≤0.25 μg/mL; resistance ≥1 μg/mL for enrofloxacin and 1 and 2 μg/mL respectively for marbofloxacin). On day 7, 18 PM isolates were obtained, each with similar or lower MICs to those on day 0. Comparable data for breakpoints of *H. parasuis*, and *B. bronchiseptica* have not been published.

**Table 5:** Bacterial pathogens isolated on days 0 and 7 from lower respiratory tract lesions, bronchoalveolar lavage and nasal swabs, minimum inhibitory concentrations (MIC) and susceptibilities to marbofloxacin and enrofloxacin

		Marbofloxacin					Enrofloxacin				
		MIC range	MIC_50_	MIC_90_	S%	n	MIC range	MIC_50_	MIC_90_	S%	*n*
*A. pleuropneumoniae*	Day 0	0.03–0.12	0.06	0.06	100	61	0.015–0.12	0.06	0.06	100	61
	Day 7	nc	nc	nc	nc	0	nc	nc	nc	nc	0
*H. parasuis*	Day 0	0.25–2.0	0.015	0.06	n/a	18	0.004–1.0	0.015	0.03	n/a	13
	Day 7	0.015	nc	nc	n/a	2	0.008	nc	nc	n/a	2
*P. multocida*	Day 0	0.008–0.03	0.015	0.03	100	32	0.004–0.015	0.008	0.015	100	32
	Day 7	0.008–0.03	0.015	0.03	100	18	0.008–0.015	0.008	0.008	100	18
*B. bronchiseptica*	Day 0	0.25–1.0	0.5	0.5	n/a	92	0.25–0.5	0.5	0.5	n/a	92
	Day 7	0.25–0.5	0.5	0.5	n/a	138	0.25–1.0	0.5	0.5	n/a	134

MIC_50_, MIC_90_, lowest concentrations (μg/mL) of the antimicrobial for which 50 and 90% of the isolates were inhibited, respectively; n, total number of isolates from lung/lower respiratory tract, bronchoalveolar lavage and nasal swabs, combined, for which MIC was determined; n/a susceptibility data not available; nc, not calculated for <10 isolates; S, percentage of isolates within a species that were susceptible to the antimicrobial based on European susceptibility monitoring and where available CLSI breakpoints [28, 29, 38]

## Discussion

In the present study, the clinical efficacies of marbofloxacin and enrofloxacin were similar and were characterised by rapid reductions in clinical signs and pyrexia as indicated by marked reductions in individual clinical signs two days after treatment and, by day 7, clinical signs were absent or mild in all pigs and mean temperatures for each treatment group were <39.5 °C. However, reductions in rectal temperatures (which may occur in the absence of resolution of clinical signs) were not considered alone and were interpreted in conjunction with clinical signs when determining clinical cure. The analysis of the primary efficacy criterion, percentage of animals with clinical cure at day 7, confirmed the efficacy of the antimicrobials in the treatment of SRD and actinobacillosis and the non-inferiority of marbofloxacin compared with the reference antimicrobial, enrofloxacin. Clinical cures for each antimicrobial on days 7 and 21 were similar, (81-84%) and the percentages of animals (3.5–5.0%) that either relapsed after day 7 or where removed at any time from the study for SRD reasons were also similar.

Comparison of these efficacy results with those of previous studies should be made circumspectly because of likely differences in, for example, aetiology, animals, environment, antimicrobial sensitivity of the pathogens, clinical severity at time of treatment and efficacy criteria. This makes it difficult to reach conclusions regarding the relative efficacy of different antimicrobial classes in the treatment of APP and SRD. The results of this study suggest when there is no non-critical alternative antimicrobial available and relevant epidemiological and sensitivity results are supportive, that use of one of the injectable fluroquinolones, marbofloxacin or enrofloxacin may be anticipated to provide efficacy in pigs with acute clinical APP. The efficacy results in the present multicentre study for the treatment of SRD associated predominantly with APP were comparable to the results obtained in other field studies. For example, in an outbreak of SRD and actinobacillosis treated with amoxicillin (7 mg/kg/day) or marbofloxacin (2 mg/kg/day) each administered daily for 3–5 days the clinical cures on day 5 were 68 and 74.5%, and relapses to day 21 of 11.9 and 17.2%, respectively [34]. In that study, each of the dosage regimes facilitated time-dependent bacterial killing which, it is now known is appropriate for amoxicillin but is less effective when used for fluroquinolones that are more effective when given in higher doses to facilitate concentration-dependent bacterial killing [6, 7]. In this field study, dosage regimens appropriate for concentration-dependent bacterial killing were administered. For both marbofloxacin and enrofloxacin, efficacy was assessed based on clinical efficacy rather than on post-treatment bacteriology. In two, single-centre, studies conducted in USA on SRD in pigs with mixed infections of APP, PM, *H. parasuis* and *Streptococcus suis*, the efficacy 4 days after treatment with a single dose of enrofloxacin 7.5 mg/kg was 33.0 and 89.7%; however, details of mortalities and subsequent relapses were not given [35]. In another report of six field studies conducted to a common protocol in five geographically separate centres in USA and Canada, the overall efficacies for treatment of SRD by tulathromycin and ceftiofur were 70.6 and 64.4%, respectively, and the efficacies for treatment of SRD predominantly associated with APP were, in Iowa, 68.2 and 79.5%, respectively, and in Nebraska, 81.3 and 77.1%, respectively [18]. In another multicentre, field study of outbreaks of SRD conducted on farms in Germany, France, UK and Netherlands, in which the efficacy criterion was animals successfully completing the study 10 days after treatment, efficacy was 82% in tulathromycin-treated pigs and 68.4% in pigs treated with either tiamulin or florfenicol [36].

**Fig. 1:**
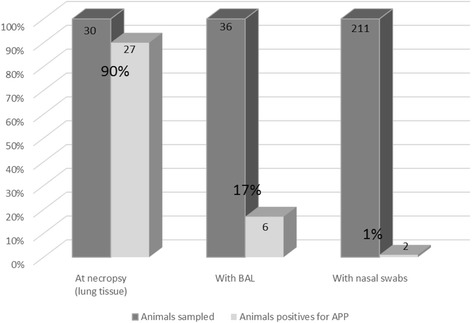
Percentage of APP isolation from different sampling sites at inclusion to the study

In this study, necropsies of clinically affected pigs were conducted immediately prior to beginning treatment on each affected farm and showed gross pathologic lesions of diffuse fibrinous pneumonia, typical of acute actinobacillosis. The principal and numerically predominant pathogen isolated from lung tissues at necropsy was APP as confirmed by PCR. Based on the bacteriology of the lung and BAL samples, it is possible that some of the individual cases of actinobacillosis were complicated by secondary, concurrent infections of PM*, H. parasuis* and/or *B. bronchiseptica*. In pigs, successful isolation of pathogens associated with active pneumonia from BAL and nasal samples varies between bacterial species and, particularly for APP the frequency of isolation tends to be low [20, 37]. In the present study, distribution of APP isolates at inclusion indicated that lung sampling at necropsy was the most accurate technique to isolate this pathogen compared with BAL and nasal swab (Fig. 1). There were large differences in APP isolation frequency between the sampling methods suggesting that sampling of the upper airways in pigs may not allow the correct identification of the causal pathogen(s) of lower respiratory tract bacterial infections and thus potentially misleading the choice of antimicrobial for treatment.

The MIC_90_ values for APP isolated from the necropsy and BAL samples was 0.06 μg/mL for each of the antimicrobials and were similar to those reported in an European antimicrobial susceptibility monitoring survey of isolates collected from untreated clinical cases in 2002–2006 [38]. This and other reports [39, 40] suggest that there had been little or no change in susceptibility of APP European field isolates to marbofloxacin and enrofloxacin between 1994 and 2009. Comparison of the MIC values of marbofloxacin and enrofloxacin determined in the present study with the values in the European survey for APP and PM indicated that all of the isolates of these species would have been susceptible to both of the antimicrobials. In vitro, APP is typically susceptible to a wide range of antimicrobials including fluoroquinolones although there is increasing resistance to penicillins, tetracylines and trimethoprim-sulphonamides [2, 38–40] and normally these or other non-critical antimicrobials should be used in preference to fluoroquinolones and consistent with antimicrobial usage policies and product labels [14, 15].

The variability in PK parameters between animals and the variability in PD parameters (e.g. MIC) within populations of microorganism may influence efficacy and the potential for resistance development in the target pathogen. Simulations of this variability in PK-PD have been used to evaluate marbofloxacin in the treatment of APP infections in nursery and fattening pigs, and showed that a single dose of 8 mg/kg would provide robust efficacy and minimise resistance development in APP with MICs of 0.03–0.12 μg/mL [9, 10] which are comparable to the APP MIC range reported here.

Limitations of this multicentre clinical trial include the following. The number of individual farms, four was small and may not represent a wider diversity of naturally occurring SRD outbreaks with differences in pathogens, environment, husbandry, pig genotypes and timing of treatment interventions. The individual farm outbreaks of disease were not on their own large enough to enable evaluation of farm (or outbreak) by treatment interactions. The primary efficacy variable (cure on day 7) was based on discontinuous and subjective clinical scores whereas preferably it would use a larger number of independent, continuous and objective variables. Pyrexia as measured by rectal temperatures is an objective and continuous variable however on its own it may be misleading as pyrexia may resolve without cure necessarily having occurred. This study used a positive control whereas scientifically a negative control study may be preferred however, this would be expected to raise ethical and animal welfare concerns. The efficacy cure rates observed in this and other published studies may not be replicated exactly under different clinical conditions.

## Conclusions

Marbofloxacin treatment as a single IM dose of 8 mg/kg was clinically safe and clinically effective in the treatment of respiratory disease associated predominantly with APP in four European commercial, fattening pig herds. Enrofloxacin given either as 1 or 2 doses of 7.5 mg/kg was also safe and effective.

## Abbreviations

APP: *Actinobacillus pleuropneumoniae*
BAL: Bronchoalveolar lavage
CLSI: Clinical and Laboratory Standards Institute
CP: Control product
IVP: Investigational veterinary product
MIC_50_: MIC_90_, lowest concentrations (μg/mL) of the antimicrobial for which 50 and 90% of the isolates were inhibited, respectively
MPC: Mutant prevention concentration
PD: Pharmacodynamics
PK: Pharmacokinetics
PM: *Pasteurella multocida*
SRD: Swine respiratory disease
